# Implications of an Autism Spectrum Disorder Diagnosis: An Interview Study of How Physicians Experience the Diagnosis in a Young Child

**DOI:** 10.3390/jcm7100348

**Published:** 2018-10-12

**Authors:** Delphine Jacobs, Jean Steyaert, Kris Dierickx, Kristien Hens

**Affiliations:** 1 Center for Biomedical Ethics and Law, KU Leuven, 3000 Leuven, Belgium; kris.dierickx@kuleuven.be; 2 Center for Autism Expertise, UPC-KU Leuven, 3000 Leuven, Belgium; jean.steyaert@uzleuven.be; 3 Department of philosophy, University of Antwerp, 3000 Leuven, Belgium; kristien.hens@uantwerpen.be

**Keywords:** autism spectrum disorder, diagnosis, physicians, interviews, interpretative phenomenological analysis, experiences, psychological consequences, relational consequences

## Abstract

Clinicians are significant translators and interpreters towards parents of the abundant literature on autism spectrum disorder (ASD). However, how clinicians experience and view ASD and an ASD diagnosis is not well known. Sixteen physicians working with young children with a (presumed) diagnosis of ASD participated in a semi-structured interview. They described their professional view on ASD and an ASD diagnosis, and how they experienced its use in their clinical practice. Interpretative phenomenological analysis of the data revealed two main topics about physicians’ experiences: how they view ASD and an ASD diagnosis, and how they experience the implications of an ASD diagnosis. The latter topic comprised three themes: (1) the ASD diagnosis leads to a particular treatment trajectory and services; (2) ambivalence about how the ASD diagnosis impacts parents and child; and (3) psycho-relational functions of the ASD diagnosis. Physicians feel that some doubts and questions are inevitable when dealing clinically with ASD and an ASD diagnosis. They also perceive that there are certain risks associated with assigning the categorical ASD diagnosis to a young child. Altogether however, ASD is perceived by physicians as a useful and valuable diagnosis both because of treatment related consequences and of several psycho-relational implications.

## 1. Introduction

Autism spectrum disorder (ASD) has gained much attention in recent years, as increasingly more children are receiving an ASD diagnosis. Globally, 1 in 160 children is estimated to have ASD, and in the US, estimates of ASD’s prevalence are even higher, at 1 in 59 among eight-year olds [1]. ASD is understood to be a neurodevelopmental condition, one characterized by early-onset difficulties in social communication and interaction, and unusually restricted, repetitive behaviour and narrowly focused interests [2]. ASD is diagnosed clinically, often in a multidisciplinary assessment assisted by specific diagnostic tests [3]. Rather than the picture becoming clearer after several decades of research, a consensus has emerged that ASD is a complex and heterogeneous disorder [4]. This is the case in terms of its phenotypical presentation and with regard to aetiology, treatment, and prognosis [5,6,7]. Such complexity and heterogeneity can prove problematic for clinicians, who have the responsibility of applying a diagnosis of ASD to a child, or of treating him/her and his/her parents. 

It is known that receiving a diagnosis of ASD greatly affects the child and his/her parents: indeed, the diagnosis can yield some beneficial effects [8,9], but it can also be associated with risks, disadvantages, and contraindications [10,11,12]. Concerning the consequences of an ASD diagnosis, both parents and clinicians expect an effective intervention once a diagnosis is given [7]. An ASD diagnosis can inform the clinician on what advice on the educational approach might be useful to parents [13]. Also, extensive support at home and at school is provided in most Western countries, which is often not the case for other psychiatric diagnoses [14]. For example, in Flanders, Belgium, the Flemish Agency for People with a Disability provides extensive services for intellectually disabled (ID) people and people with ASD, but not for other chronic or life-long Diagnostic and Statistical Manual for mental disorders (DSM)-diagnoses [2], as by this government institution both ID and ASD appear to be considered disabilities, whereas other DSM diagnoses are considered psychiatric conditions, which should be professionally guided within psychiatric institutions [15].

Besides advice and support, an ASD diagnosis can also lead to ASD-specific individual interventions. Concerning pre-school children, there has been an increasing number of published trials on such intervention programs: a meta-analysis of 32 studies (about what the authors define as behavioral, social-communication focused and multimodal-developmental intervention models) did not find enough evidence that early interventions for children with ASD reduce their autism severity [16]. However, the authors detected two promising targets for these interventions: ‘reciprocity of social interaction toward others’ and ‘parental synchrony’. In general, progress has been noted in this domain, although a long journey remains ahead and many challenges are still posed, between others due to the limitations in the research undertaken [17,18].

Thus, an ASD diagnosis can lead to enrolment in a guidance or intervention trajectory. Also, psycho-social benefits to parents like an explanation and a legitimization have been found [19,20]. Although an ASD diagnosis implies several of these psycho-social benefits, it also carries some risks: psychological risks like elevated parental stress, social risks like stigmatization, and relational difficulties in the parent–child relationship [11,21,22]. A definitive weighing of benefits and risks of the clinical use of an ASD diagnosis has not yet been done. However, it has already been argued that a clinical approach based on the findings from basic ASD research [23] may be only partially helpful in responding to parents’ worries about and problems with a mildly impaired child [10,11,24,25]. A seeming disconnect exists between the mainstream scientific research agenda and the immediate priorities of the clinical and ASD communities, for example knowledge gaps and research priorities identified by researchers may be different from those identified by other stakeholders [26]. Moreover, it is unclear how clinicians experience and understand ASD and perceive the consequences of an ASD diagnosis since the concept is found to be heterogeneous and changing [27,28], and research findings on ASD are diverse and not always consistent [18]. Additionally, it is known that clinicians’ view on behavioral problems influences their clinical work with these in a significant way [29,30,31]. 

In sum, how clinicians actually experience ASD and feel about the implications of an ASD diagnosis, and especially how these views influence their response to parents who ask for help with their child with ASD-like behaviors is important knowledge, yet has rarely been studied [32]. Physicians’ experiences diagnosing ASD have already been investigated with different purposes e.g., through an online questionnaire exploring physicians’ opinions on key areas of service [33]. However, our aim is different i.e., to explore physicians’ experiences and understandings of ASD and of an ASD diagnosis. 

In this paper, we take a step toward filling this gap. The goal of this qualitative study is to explore and gain an insight into the way clinicians experience ASD and an ASD diagnosis in their clinical practice. Indeed, clinicians constitute an essential passage in the transfer, translation and interpretation of ASD research findings to the lay public and particularly to stakeholders, i.e., persons with a diagnosis and their parents. We confine our study population to physicians working with young children without intellectual disability (ID) or other disability. To better appreciate physicians’ experiences with ASD and the diagnosis of ASD, we also investigated how physicians perceive the implications and impact of an ASD diagnosis. That is, how the diagnosis affects doctors, the parents and the child with a (presumed) diagnosis. Our findings on physicians’ views on ASD (diagnosis) will be presented separately in another publication [34]. Here, we discuss the implications of an ASD diagnosis as experienced by physicians. 

## 2. Experimental Section

### 2.1. Sample

Although many types of healthcare professionals work with children with a (presumed) diagnosis of ASD, we chose to include as participants a select group of physicians in Flanders, Belgium, who work with particular children. This interview study included only physicians who were (at least partly) working with children younger than six years of age, without intellectual and other disability, and with a diagnosis of ASD or who displayed ASD-like behaviors that led parents to ask for an ASD diagnostic assessment. The group of physicians working with these children is of particular interest, because the increase in prevalence of the diagnosis in the last decade is partly attributed to children without comorbid ID being more often diagnosed [2], and because recently there has been a growing emphasis on diagnosing children with suspected ASD early on [8]. The physicians we included are involved early on in the assessment and treatment of young children with a (presumed) diagnosis. There are specific ethical questions that arise concerning the use of an ASD diagnosis in young children. For example, there is debate about whether research should focus on identifying cures for early-diagnosed ASD [23], and questions can be asked about the ethical implications of the only moderate stability of an early ASD diagnosis in preschool children [35]. Since these children were not intellectually or otherwise disabled, the diagnosis of another handicap arguably would not ‘overshadow’ a physician’s experiences of the diagnosis of ASD in these children. We confined our sample to the profession of physicians in order to obtain a homogeneous group [36], and because in Flanders, a physician is necessarily a part of the multidisciplinary team performing ASD assessments. Such a team does a clinical assessment in different developmental domains, most often assisted by diagnostic tests -some of which are evidence-based, like ADOS (Autism Diagnostic Observation Schedule) and WPPSI-R (Wechsler Preschool and Primary Scale of Intelligence–Revised). Concerning post-diagnostic support, Flemish physicians may provide coordination of the professional help (especially at school), parent guidance, developmental follow-up of the child, and psychotropic drug prescription and follow-up; often they delegate (parts of) this support to other clinicians. Candidate physicians were purposively sampled [37] to be participants. They were contacted by an e-mail through two professional physicians’ organizations: the Flemish Association of Child and Youth Psychiatrists (VVK) and the Flemish Association of Disability Physicians (VVAG). We chose these two organizations, because for children in Flanders younger than 12 years, ASD is most often diagnosed in those who visit: specialized outpatient clinics (58.2%), or rehabilitation centers (21.4%), or who are seen by child psychiatrists (13.3%) and pediatricians (2.0%) [38]. The authors of this study do not specify how they defined a “specialized outpatient clinic”, and do not report which specific types of specialized physicians were working in specialized outpatient clinics and rehabilitation centers. In our experience, in these two types of centers child psychiatrists, child neurologists, disability physicians, and pediatricians are working with i.a., the clinical population of this study, i.e., children younger than six years of age without disability. Fourteen physicians responded to our e-mail, agreeing to be interviewed. After these 14 interviews were completed, we complemented the sample of 14 volunteering physicians by explicitly inviting two more child neurologists to participate, in order to broaden the data set to include experiences of different types of physicians who regularly work with these children in Flanders [39]. Thus, the final purposive sample comprised 16 physicians, of whom 9 were child psychiatrists, 4 were child neurologists, 2 were disability physicians, and 1 was a pediatrician (Table 1). Information about the study was given orally and in written form, and all persons gave their informed consent prior to their inclusion in the study, and to the publication of these study results. The Ethics Committee of the University Hospitals Leuven approved this study on 3 Page: 3 February 2017 (Belgian Registration Number B322201731147). This study was performed in accordance with the ethical standards laid down in the 1964 Declaration of Helsinki and its later amendments. 

### 2.2. Data Collection

Qualitative data were gathered by using semi-structured one-to-one interviews. We based the topic guide (Document S1) for the in-depth interview on our findings from a literature review on how clinicians conceptualize ASD [34,40]. 

Since we used the qualitative method of interpretative phenomenological analysis (IPA) [41], the interviews focused on gathering information about how physicians experience ASD and an ASD diagnosis professionally. In expressing their experiences, physicians were invited to describe both emotions and thoughts. Indeed, the content of one’s thoughts contributes to the phenomenological experience of emotion, and accounts for differences in the experience of cognitively complex emotions [42]. The interview guide consisted of open-ended questions. It was used openly and flexibly, in order to encourage the participants to elaborate freely and reflectively on a theme they wanted to discuss, even if that theme would be covered later in the interview. Regardless, the same content was covered with each participant. At the end of the interviews, the participants were asked if they wished to express anything else about the topic. All interviews were conducted in Dutch by the first author (D.J.) and audio-recorded. Data were collected between July 2017 and February 2018. The interviews mostly took place at the participants’ offices, a few times at the researcher’s office, or at the participant’s home. Interviews lasted for 40 to 100 min. In only two cases the interviewer noticed time pressure affecting the interviewee. Participants were free to say more on an interview topic if they chose to, and some physicians used more time than others to clarify their experiences.

### 2.3. Analysis

The data were analyzed using the procedures outlined for IPA [41]. Briefly, IPA employs inductive analysis, grounded in the material, and in the second place a dialog of the resulting findings with already existing theories. Smith and colleagues talk of a “double hermeneutic” in which the interviewees give interpretative narratives of their experiences, and the researcher analyzing these narratives interprets them when identifying themes that belong to the domain of the research question [41]. 

D.J. and the last author (K.H.) listened to the recordings independently. D.J. transcribed each recorded interview verbatim, and then systematically and inductively coded the transcripts line-by-line in NVivo 11 (QSR International, 2017). The goal was first to identify thematic passages of text across the data of one interview. Consequently, a comparison was made across interviews. The analysis was collaboratively conducted by D.J. and K.H. In addition, all authors met regularly to discuss the interviews and the codes. The primary codes were clustered into recurrent subthemes and synthesized into themes (Table 2). Finally, an overall narrative account was created through a group analysis of the different interviews. Representative quotations were translated verbatim from Dutch to English, in order to illustrate the original expressions. Square brackets indicate information added from the interview material before or after the quote in order to give context for the quote.

Several strategies were employed to strengthen the validity of the findings. First, the authors frequently met and discussed the data and their analysis. Second, the research project was subjected to peer scrutiny by researchers from the local psychology faculty and of the pedagogy faculty. Finally, the researchers continually maintained a reflexive stance [43]. These deliberative procedures enhanced the trustworthiness of the findings. 

The anonymized data used and analyzed in this study are available from the corresponding author on reasonable request.

## 3. Results

Physician participants’ ages ranged from 30 to 65 years (Table 1). Most of the physicians were female (75%), which is in line with the gender distribution in these professions in Flanders (Gemengde Werkgroep Psychiatrie, kinderen en jeugd. 23 June 2016. Consulted at 21 March 2018, http://overlegorganen.gezondheid.belgie.be). The work settings for participant interviewees were diverse, but most were practicing in a hospital or in a center for developmental disorders.

Our IPA analysis of interview transcripts revealed many codes related to physicians’ experiences with the implications of an ASD diagnosis. Within this topic we identified three main themes: (1) the ASD diagnosis leads to a particular treatment trajectory and services; (2) ambivalence about how the ASD diagnosis impacts the parents and child; and (3) psychological and relational functions of the ASD diagnosis (Table 2). Next, we will discuss these themes consecutively and present representative quotations of the physicians (in italics) to illustrate the theme.

### 3.1. Theme 1: ASD Diagnosis Leads to a Particular Treatment Trajectory and Services

Characterizing physicians’ experiences with ASD and a diagnosis of ASD was complex. They said that ASD could be understood in several ways. Accordingly, most physicians reported feeling that it is hard to know what actually constitutes ASD. They also found it difficult to assign a diagnosis to a child and to explain it sufficiently well to his/her parents. Most physicians were convinced that an ASD diagnosis should be given only if it would lead to useful advice on a more effective approach to educate the child (both at home and outside the home). Indeed, all physicians stated that one of the possible benefits of an ASD diagnosis is that it guides the therapy course, such as which psycho-educational advice to give to parents. For example, this physician expresses that psycho-educational advice about the need for an autism friendly structured approach is very useful:

But the biggest advantage [of an ASD diagnosis] is advice towards an [educational] approach [to use], a more structured approach.

—(Physician 9)

Hence, the usefulness of the diagnosis lies in reference to a workable approach for education. Once an ASD diagnosis is given, almost all physicians adhere to what they believe to be the “standard” approach. That is, the work to achieve what children with an ASD diagnosis are found to need from their environment: structure, predictability, and (less frequently mentioned) visualization (i.e., using images and pictures to explain a situation and the environment’s demands to the child). At the same time, many physicians felt that these measures are also useful for other children, without an ASD diagnosis.

In fact, an ‘auti-approach’ is helpful for a lot of children with whatever problem [they have]. I think it provides peace for a great deal of children.

—(Physician 2)

Thus, our analysis suggests that these physicians feel that a diagnosis of ASD guides the specific kind of approaches to be tried with a child with a diagnosis. Nevertheless, the ‘standard’ ASD approach (one that mostly provides structure and predictability) is regularly not perceived as being so specific that it would be inapplicable to children who display behavioral problems but are not diagnosed with ASD. 

Our participants also felt that this ‘standard’ approach for diagnosed children always needs to be supplemented with personalized treatment. That is, physicians should search for what ‘works’ for that particular child and in the child’s specific environment. In this search for a beneficial approach, physicians valued the parents’ experience with the child and the parent–child relationship, and, to a lesser extent, how the child’s behavior could be understood. This last point is illustrated in the following comment:

I propose to parents that we are going to try to understand what their child is ‘saying’ with his behavior.

—(Physician 4)

In line with their preference for using an individualized search (versus providing the ‘standard’ advice only), physicians often voiced their wish to gain an all-encompassing, descriptive picture of the child, because they do not consider a one-size-fits-all approach to be beneficial when it is based solely on a diagnosis. Indeed, they often viewed the ASD diagnosis as insufficient, since with such diagnosis in isolation, physicians felt they could not implement a satisfying guidance plan. Moreover, they felt that they could not fully understand the unique child as a whole. For example, the following physician said that before (or without) giving a classificatory diagnosis based solely on DSM criteria, she constructs a broad description of the child and of how he/she is unique:

We always look at the unique child. The diagnosis only comes to the fore secondarily, I value a descriptive diagnosis more than a DSM classification.

—(Physician 5)

Besides offering advantages for selecting the appropriate approach towards the child, these physicians emphasized that an ASD diagnosis confers important entitlements to different services that would otherwise be unavailable, for example at home and at school. Participants never mentioned an entitlement to ASD-specific child-directed interventions. In families with a diagnosed child, physicians advised parent-guidance (by the physician him/herself and by other professionals). At school, teachers can get advice on how to adapt the classroom and the teaching situation to help the child. The participants often expressed regret that such entitlements are coupled to an explicit, written diagnosis—instead of the child’s generic everyday difficulties. As the following comments demonstrate, an ASD diagnosis can be an “entrance ticket”, that opens doors:

A diagnosis is an entrance ticket. Especially if your child with autism is not intellectually disabled, you do need that [official] diagnosis.

—(Physician 8)

Of course, a diagnosis is still absolutely necessary to open a number of doors.

—(Physician 4)

As shown in the following quotation, physicians may almost feel coerced to make an ASD diagnosis because of the diagnosis-services link—to receive services one needs a written diagnosis:

We are forced to use the ASD diagnosis, to give the [child’s] problems a name, although I view it [the behavioral issue] as a developmental delay. I only use the [ASD] term to be able to communicate about it.

—(Physician 1)

Thus, even if this physician prefers to use a different way to describe the behavior of the child, current administrative structures of entitlements and services may force him to use the ASD diagnosis. 

### 3.2. Theme 2: Ambivalence about How the ASD Diagnosis Impacts the Parents and Child

In relation to the potential emotional and social impact of receiving an ASD diagnosis, physician participants strongly took into account how parents received the physician’s description of the child. They frequently stated that they thought that the ASD diagnosis had a dual effect on parents: the parents were devastated and yet relieved. In some cases, the physicians noticed how parents actively pursued an ASD diagnosis and how these parents were enormously relieved upon receiving the diagnosis. For example in the following quote, it is easy to conclude that some parents considered *not* receiving an ASD diagnosis for their child as “bad news”. This physician felt that, besides needing help, these parents needed to hear that their child’s behavior was not their fault:

In that [specialized ASD] center, I always had the feeling of bringing bad news [to the parent], if I did not give a diagnosis [of ASD, in a feedback session]. For the reasons I already gave earlier: extra help [was needed] as well as an affirmation that ‘we are not to blame; it is our child.’

—(Physician 10)

This perception is also related to the third theme (see below). Physicians in our study reported that children receiving an ASD diagnosis as well as their parents experienced some negative impact, although the physicians believed that the advantages of the diagnosis outweighed its negative implications. Physicians often said that they were acutely aware that an ASD diagnosis was *generally considered* to be ‘for life’, even if they were rarely convinced that it was correct to view ASD as a permanent condition. For example, the following participant explained how he thought ASD often improves after a certain time:

Over the course of the [childhood] years, you see the autism lighten up; it becomes less pathological. You do not know where [along the spectrum of special to average] a child finally will be.

—(Physician 1)

Also, these physicians worried about the negative effects resulting from the child being exclusively seen and treated as ‘the autistic child’. For example, this physician was concerned that every different or unusual behavior the child displays would be attributed to the ASD diagnosis:

When too much is attributed to [the view], ‘This is once again due to that autistic side of yours’, then little by little the child becomes [seen] only [through the lens of] his diagnosis.

—(Physician 10)

Besides, worrying about the potential negative effect on the child’s self-image, the following participant gave some insight into how a physician may feel when he/she tells children that they are now “officially different”:

At the time when I explain the [ASD] diagnosis to young children, I always see in these little faces—no, I rather feel it—that a doctor is officially affirming that they are different.

—(Physician 4)

This ambivalence physicians showed about the negative and positive impact of an ASD diagnosis was not restricted to young children with an ASD diagnosis. Some physicians we interviewed also were working with somewhat older children and adolescents. While some said an ASD diagnosis was mostly experienced negatively by the adolescent, others said they perceived a predominantly positive effect on an adolescent’s self-esteem. Rather than being perceived as weird by peers, as would be the case in the absence of a diagnosis, *with* an ASD diagnosis there was a formal explanation for the adolescent’s behavioral differences. This is related to the third theme on the psycho-relational functions of the ASD diagnosis. Other physicians felt that an ASD diagnosis given to a teenager put him/her on a difficult trajectory through puberty. When a teenager receives an ASD diagnosis, physicians often felt this was more consequential than receiving a diagnosis at a younger age, since adolescents are in a critical stage with regard to identity formation.

Only one physician reported having a conversation with a young child concerning the ASD diagnosis. Mostly they said that they depended on the parents to disclose the diagnosis to the child at an older age. Some physicians argued that hiding an ASD diagnosis from a child would cause problems, but rarely they mentioned what they considered to be a suitable age for telling the child, nor did they mention whom they thought should deliver the information to the child.

### 3.3. Theme 3: Psycho-Relational Functions of the ASD Diagnosis

Our participants reported experiencing other important implications of an ASD diagnosis. These effects seemed more or less consciously and willingly pursued in an ASD diagnostic process by physicians or parents although they were less directly related to subsequent care for the child. Rather, they were associated with psychological and relational implications, functions or roles of the diagnosis. Participants observed that the ASD diagnosis had four functions: (1) the diagnosis could provide a plausible and clear explanation to parents, child, and others; (2) the diagnosis could ‘prove’ to parents (and others) that their worries about the child were real and legitimate; (3) the diagnosis could reduce parents’ expectations for the child, for example, helping parents to be more understanding and patient toward the child; and finally (4) the diagnosis could lift blame from both parents and child (i.e., exculpation). 

First, many interviewed physicians felt an ASD diagnosis could provide a plausible explanation (1) to the parents, (2) to the child, and also (3) to others. The following three quotes by the respondents illustrate this effect vis-à-vis these three recipients.

*It can be a relief [for parents, if their child receives an ASD diagnosis]: “So now we have an answer.” I mean “There is an explanation for the reason why he is acting the way he does.”*

—(Physician 5)

*With this diagnosis of [ASD], several children have told me that their different way of thinking, their being interested in other things, it can now be explained.*

—(Physician 3)

*I think it is easier to explain to people, I mean, some parents are very ashamed about the behavior of their child: “You are in a shop and then he gets this screaming fit”. (…) It is helpful if they can explain to people “He really has autism, there is something that causes him to act this way”.*

—(Physician 5)

In this explanatory function, most physicians concluded that parents viewed the practical use of the label ASD and its potential connotations to be more important than it being a factual causal explanation of their child’s condition. That is, that the ASD was caused by a medical and genetic condition. Physicians felt that parents were rather superficially interested in the cause underlying ASD. Instead, they were more interested in how they could use the ASD diagnosis in a pragmatic way. This idea is illustrated in the expressions of physician 12 and 14:

A lot of people do know that it [ASD] is a family thing; they do not know exactly, but that it is heritable.

—(Physician 12)

Most parents ask about causes, but the focus rapidly becomes “And what can we do about it?”

—(Physician 14)

In the second functional aspect of the ASD diagnosis, several of the physicians valued the diagnosis because they felt it conveys to parents that their worries are real and legitimate. The quote of the following physician illustrates her experience that how giving their child an ASD diagnosis can provide to parents external recognition and validation of their problems.

*Sometimes, I meet parents who are happy with [receiving] a label of autism [for their child] because they finally—yes—get a bit of recognition of the problem they have been stuck with for a long time.*

—(Physician 6)

In the third functional aspect of the ASD diagnosis, many of the physicians said that parental expectations of their child with an ASD diagnosis decreased in two ways: (i) spontaneously, parents often expected less out of their child once he/she received the diagnosis, and (ii) with help from physicians, parents were now more open to recognizing that they could reduce the expectations of their child. Physician 4’s quote illustrates how, if the child had received an ASD diagnosis, parents might have lowered their expectations towards their child:

I have been seeing these parents [of a child without an ASD diagnosis] for many years now and the recurrent theme is: what is the balance [we should aim for] between understanding and setting limits. The father draws the line very strictly, while the mother is very understanding. So, it is not as if we did not talk about this topic, it is the common thread throughout all these years and still I think: maybe we should have given the ASD diagnosis in order to create more space for understanding in the parents, and to lower parents’ expectations.

—(Physician 4)

The quote of the following physician illustrates that a diagnosis can give parents the permission to pare down their expectations.

*Yes, I think that most of the parents say “Okay, it is okay that we lower our expectations” because I think that, by that time [when they get a formal ASD diagnosis], they are already doing this [lowering expectations].*

—(Physician 12)

At the same time, several physicians felt that an ASD diagnosis offered them the possibility to work with parents toward the goal of lowering their expectations for their child. This idea is illustrated in Physician 2’s discussion with parents about an Intelligence Quotient (IQ) test given to their diagnosed child.

*I tell parents that “this IQ [result] is not the same as it would be for a normal child, it is not showing up in the same way”. I think that most parents do learn to tell themselves: “I should not focus too much on his IQ and tell him that he should be able to do that [task]”. Yes, to lower their expectations.*

—(Physician 2)

In the fourth functional aspect of the ASD diagnosis, our participants said that exculpation was of the utmost importance for the parents. They felt it functions in two ways. First, an ASD diagnosis was experienced to serve to lift blame from the parents and the choices they made and will make in the way they educate their child. Second, it also served to lift blame from the child. For many of the physicians, the exculpatory function was one of the most important implications of an ASD diagnosis. They said that they believe parents never are to blame for the behavioral problems of their child. Several physicians reported that, when a child exhibited what some people considered to be problematic behavior (not necessarily autism-like), people blamed not only the child for the poor behavior, but also the parents for their child-rearing practices. This experience is illustrated in the following quote:

Parents very often say that people think that they [parents] are to blame [for the child’s behavior]. Even teachers regularly say “But these parents really do not provide enough direction”.

—(Physician 15)

In their clinical practice, physicians said that it is extremely important to convince parents that their child is not ‘bad’ or is not behaving ‘badly’. They want to convey to parents that their child does not have an unruly, insubordinate disposition when he/she displays challenging behavior. Physician 13’s quote illustrates that he even considers this task to be the core of his work as a clinician:

The essence of my work is making parents understand: “Your child is willing to, but is not always able to, cannot always handle it [controlling his/her behavior].”

—(Physician 13)

In the case of ASD, physicians report that a diagnosis of ASD is useful and necessary to effectively convey the exculpatory message about the child with an ASD diagnosis, i.e., that he/she is not to blame. Getting across this idea makes it possible for them to work with parents and schools, as illustrated here:

The naughty child becomes the child with a problem who is not responsible for his behavior. And for a great many of teachers that is a big difference; and so the interactions of the child are much more positive.

—(Physician 10)

Hence, our physician participants said that the exculpatory function of an ASD diagnosis was important, as it not only gives parents and children psychological relief, but also it promotes a workable and positive relationship between children, parents, educators, and clinicians.

## 4. Discussion

We interviewed Flemish physicians who were working with young children without (intellectual) disability in order to investigate their experiences of ASD and ASD diagnosis. A frequent topic in these interviews were the implications of an ASD diagnosis physicians experienced for themselves, parents and child. Overall, the participants felt that the positive implications of the diagnosis outweigh the negative ones.

With regard to the positive effects of an ASD diagnosis, our physician participants underscored the practical benefits that the diagnosis gave, specifically, in leading to professional advice and help inside and outside the parents’ home. However, some previous research shows that parents often feel that this advice and help is absent or unsatisfactory; they appear to value post-diagnostic support most [24,44]. This means that the parents prefer it when the same clinician giving the diagnosis also provides support afterwards. The participants felt that what they call ‘standard’ measures (perceived as structure, predictability and to a lesser extent visualization to the child) in ASD care approaches are useful, but also for non-ASD children, in general. The participants never mentioned ASD-specific child-directed interventions (evidence-based or not), but it is noteworthy that such child-directed treatment modules (like applied behavioral analysis and early intensive behavioral intervention) are not widely available in Flanders. The physicians were convinced that the perceived ‘standard’ measures of structure and predictability need to be supplemented with a personalized search of an approach geared towards the unique needs of the individual child. They believed that the diagnosis was an ‘entrance ticket’ to previously unavailable services for the parents and child, and sometimes felt virtually coerced to make a formal ASD diagnosis in order for parents to gain access to these services. This practice of granting an ASD diagnosis in the face of diagnostic uncertainty, strictly for the purpose of obtaining services for the child, frequently occurs in Australia: like in Flanders, in some states in Australia, access to funding for care and treatment of patients is based on the medical diagnostic category [45]. In our study, several physicians stated that they regret that a formal diagnosis is required in order to access services. This opinion is in line with mental healthcare trends, in general, where a personalized ‘clinical-case formulation’ is recommended in order to identify a broad range of idiosyncratic aspects of a clinical case and to select appropriate interventions [46]. Moreover, in France, for example, adapted services are available without a diagnosis. A description of the symptoms by professionals is sufficient to gain access to help and services [20]. In a 2013 Belgian science-policy advisory report on ASD in young children, the Superior Health Council distinguished categorical from action-oriented diagnoses, but no participant in the present study mentioned this possibility. (Advies van de Hoge Gezondheidsraad 8747, Levenskwaliteit van jonge kinderen met autisme en hun gezin. 2013. Consulted at 30 July 2018, https://www.health.belgium.be).

Our participants rarely reflected spontaneously on perceived negative effects that potentially could emerge after a classificatory ASD diagnosis. In general indeed, the process of classification is often considered fundamental (and thus positive) to any science. In medical practice, a particular diagnosis is viewed as leading to a particular cause and cure [47,48]. It is usually beneficial to treat the root cause of a disease—for example, removing an infectious agent. Much of the success of modern medicine is based on the disease model [49], where a specific diagnosis identifies a specific cause. In psychiatry however, current evidence underscores that a diagnostic category can have very heterogeneous causes [48]. Nevertheless, psychiatric diagnoses are also considered useful in clinical practice, for example when choosing a cure or giving a perspective on prognosis [50]. Moreover, on the social level, lack of a diagnosis means no visibility, no recognition, and no (medical) explanatory narrative [51]. Indeed concerning ASD, our physician participants reported that they saw the utility of an ASD diagnosis.

In the second main theme, our interviewed physicians reported experiencing the emotional and social impact that parents and children feel after getting an ASD diagnosis. The physicians often consider the impact they professionally have on the feelings of parents, and take into account how parents might receive the information about their child. Indeed, in previous research on ASD clinical practice, the importance of building a positive parent–clinician relationship has been emphasized, of clinicians being sensitive to the needs of parents and of their focus on helping the family cope with their child. This is contrasted with a clinical approach in which the focus is only on diagnosing and treating the child [25,52]. A few of our participants also said they worried about how the ASD diagnosis would potentially affect the young child’s future. The physicians we interviewed were acutely aware that a diagnosis of ASD is considered, in general, to be ‘for life’ [8,25]. In the short term however, they strongly invested in providing the child with all the support available inside and outside the home, and in freeing the child of blame vis-à-vis his/her parents and teachers.

Related to this last point and as our third main theme, we found that our physicians felt that parents experience several psychological and relational implications of an ASD diagnosis, specifically, explanation, legitimization, reduced expectations, and exculpation of parents and child. 

First, our participants felt that an ASD diagnosis was valuable in that it offered an explanation to parents about their child’s behavior. This experience is consistent with the general notion that a diagnosis is a starting point, the foundation from which sense-making and experiences are crafted [53]. Human beings have the propensity to name things, “in order to reduce the uncertainty and to offer a (more often false) sense of control, by splitting a chaotic experience into manageable chunks” [54]. In this respect, several authors warn against reification [55]: an all-too human quality in which a descriptive diagnosis becomes an explanation, “whereby a diagnostic label that is originally used to describe a set of ‘abnormal’ behaviors may come to be used as an explanation for these behaviors” [19,55]. In our society, indeed, explanations are deeply valued [56], this is also clear in our results. 

As a second psycho-relational function of the ASD diagnosis, our participants noticed that an ASD diagnosis legitimized parents’ worries about their child. Indeed, it has been known for several centuries that a *medical* diagnosis gives problems a quite different status [54]. Today moreover, the view is common that the diagnosis of a *neurological* disorder can prove to parents and others that the parents’ worries are legitimate [57]. 

As a third psycho-relational function, an ASD diagnosis can decrease parental expectations towards the child, both spontaneously and under the influence of the physician. Physicians often appreciate this consequence, although several physicians in our study also worried about children being ‘reduced’ to their ASD diagnosis. One way of reducing children to their diagnosis, could be, for instance, parents and other adult caregivers (like teachers) expecting less from a child in every single domain of their development [58]. 

As a fourth psycho-relational function of an ASD diagnosis, our respondents said they valued its exculpatory function. By assigning the child’s problems to biology, or putting them in the realm of failed normal neurodevelopment, the diagnosis is felt to lift blame from the parents. Philosopher of science Ian Hacking states: “One of the great moral benefits of ‘biologizing’ is that it relieves a person of responsibility” [59]. Evidence shows that perhaps the only positive consequence of biomedical explanations for psychopathology is their capacity to reduce the blame and personal accountability attributed [60]. Indeed, an ASD diagnosis is often not physically visible, so the exculpatory effect of ASD as a biomedical diagnosis might be experienced by our clinicians as being especially valuable. 

In synthesis of this theme of psychological and relational functions of a diagnosis, these physicians appeared to feel that, by using an ASD diagnosis in their practice, they offered “a way out” from parental responsibility and blame (i.e., retrospective responsibility, Björnsson & Brülde in [61]). The medical diagnosis of ASD seems to function to “biologize” the problem, rendering it non-discreditable and free of responsibility and blame [62]. 

## 5. Conclusions

In sum, the interviewed physicians working with young children without ID, feel that the positive implications of an ASD diagnosis overshadow possible negative effects. The practical consequences of a diagnosis are clearly valued, although physicians doubt whether a categorical diagnosis is necessary to lead to service entitlements and to advice on the educational approach of a child with autism-like behaviors. When it comes to the impact of an ASD diagnosis, physicians are strongly aware of the parents’ feelings when they consult them. Concerning the child, they mostly wish on one side to provide him/her with the services a diagnosis entitles him/her to and on the other side to exculpate him/her. Indeed finally, with regard to the psychological and relational functions of a diagnosis, particularly this exculpatory function of a diagnosis is remarkable. An ASD diagnosis is experienced by physicians as lifting blame from the parents and the child both for themselves and towards the outside world. That is, physicians find this effect useful because of the psychological relief in parents and child, and also because of the exculpation of parents and child towards others. Moreover, with parents who come to them for help with worries and problems concerning their young child, they appreciate this effect in performing their clinical and therapeutic work. 

Our study had several limitations. This interview study was conducted with physicians who volunteered to participate. Thus, there may have been selection bias. All candidates were seasoned clinicians and often eager to talk about their view on ASD. Since this is a qualitative study, we did not aim to quantify the opinions of our participants. However, with an appropriate contextual translation, the results can shed light on clinical ASD work in other regions and cultures. In addition to interviewing physicians working with young children, we are also interviewing parents of young children with an ASD diagnosis. Of course, a valuable next step would be to query persons (i.e., older children, adolescents, and adults) with a diagnosis themselves. 

Physicians experience that questions and doubts are inevitable when dealing clinically with ASD, but altogether, the positive effects of an ASD diagnosis are felt to overrule the negative effects, and in the current constellation of clinic, research and society ASD is experienced as a useful diagnosis. While the positive effects of an ASD diagnosis often depend on the social–cultural and political–economic climate [63], our study shows that, in the present climate, an ASD diagnosis is valued by physicians using it in their practice. We conclude that physicians experience an ASD diagnosis as predominantly useful and valuable in their clinical practice, because of both its treatment-related consequences and its psychological and relational implications.

## Figures and Tables

**Table 1:** Demographic and professional characteristics of study participants.

	Profession	Work Setting	Sex	Age Range (year)
1.	Child psychiatrist	Private	M	60–65
2.	Child psychiatrist	Special boarding school	F	60–65
3.	Pediatrician	Center for developmental disorders	F	40–50
4.	Child psychiatrist	Hospital (ambulatory)	F	40–50
5.	Child psychiatrist	Ambulatory center	F	30–40
6.	Child neurologist	Center for developmental disorders	F	50–60
7.	Disability physician	Flemish Fund for Disabled People	F	60–65
8.	Child psychiatrist	Hospital	F	30–40
9.	Disability physician	University	M	60–65
10.	Child psychiatrist	Hospital + private	F	30–40
11.	Child neurologist	Special boarding school + private	F	40–50
12.	Child psychiatrist	Ambulatory center + private	F	40–50
13.	Child psychiatrist	Private	M	40–50
14.	Child psychiatrist	Hospital	F	50–60
15.	Child neurologist	Center for developmental disorders	F	60–65
16.	Child neurologist	Center for developmental disorders	M	40–50
	9 child psychiatrists 4 child neurologists 2 disability doctors 1 pediatrician	4 Centers for developmental disorders 4 Hospitals 2 Private 2 Special boarding schools 2 Ambulatory centers 1 Flemish Fund for disabled people 1 University	25% Male	median 40–50

F: Female; M: male.

**Table 2:** Physicians’ experiences of the implications of an ASD diagnosis.

Themes	Subthemes
An ASD diagnosis leads to treatment trajectory and services	Advice on approach: Diagnosis not sufficient–preference for descriptive diagnosis Standard measures, also useful for other children–personalized search–parental experience and understanding child’s behavior
	Entitlements to services: Diagnosis as an entrance ticket Diagnosis as a coercion
Ambivalence about how the ASD impacts parents and child	Impact of diagnosis on parents: Dual effect (devastation and relief) Diagnosis sometimes actively pursued
	Impact of diagnosis on child: Diagnosis “for life” Exclusively seen as “autistic” Self-image Disclosure
Psycho-relational functions of the ASD diagnosis	Explanation
	Legitimization
	Reduction of expectations
	Exculpation

## Supplementary Materials

The following are available online at http://www.mdpi.com/2077-0383/7/10/348/s1, Document S1: topic guide for the interview.
