# Supplementary material for: Implications of an Autism Spectrum Disorder Diagnosis: An Interview Study of How Physicians Experience the Diagnosis in a Young Child
Source: J Clin Med. 2018 Oct 12;7(10):348. doi: 10.3390/jcm7100348 (PMC6213203; doi:10.3390/jcm7100348)
Supplement: Supplementary file 1 [file jcm-07-00348-s001.pdf]

Document S 1. Topic guide for interviews: Physicians' experiences with Autism Spectrum Disorder (ASD) and with an ASD diagnosis

RESEARCH QUESTION
How do physicians experience ASD and an ASD diagnosis?

1. Can you describe how you view ASD? What does ASD mean to you?

   How do you understand ASD?
   How do you feel about the way ASD is thought and talked about in general (in our society)?
   - Neutral, "positive," "negative"?

2. What do you think and feel before, during, and after a diagnostic ASD assessment (by yourself or others)?

   Can you describe what you think and feel ***before*** a diagnostic ASD assessment?
   Can you describe what you think and feel ***during*** a diagnostic ASD assessment?
   Can you describe what you think and feel ***after*** a diagnostic ASD assessment?

3. Can you describe how you experience an ASD diagnosis?

   How do you experience receiving an ASD diagnosis for child/parents?
   - What, in your experience, happens when/after a child receives an ASD diagnosis (to yourself, child, parents)?
   - Neutral, "positive", "negative"?

   Impact of an ASD diagnosis on the life of child/parents?
   - Has the life of child/parents changed after receiving a diagnosis?
   - What exactly changed, if anything?

   Impact of an ASD diagnosis on how you/child/parents look at the child?
   - Your look at the child?
   - The look of the child at herself?
   - The look of the parents at the child?
   - What exactly changed, if anything?

Are there things that are important to you that have not yet been addressed or things that I have forgotten to ask? Would you like to add something or change an answer?
